# A Simple Risk Score Based on Routine Clinical Parameters Can Predict Frailty in Hospitalized Heart Failure Patients

**DOI:** 10.3390/jcm10245963

**Published:** 2021-12-19

**Authors:** Marta Kałużna-Oleksy, Agata Kukfisz, Jacek Migaj, Magdalena Dudek, Helena Krysztofiak, Filip Sawczak, Magdalena Szczechla, Katarzyna Przytarska, Ewa Straburzyńska-Migaj, Marta Wleklik, Izabella Uchmanowicz

**Affiliations:** 1 1st Department of Cardiology, Poznań University of Medical Sciences, 61-848 Poznań, Poland; agata.kukfisz@gmail.com (A.K.); protozoa@tlen.pl (J.M.); magdamroz8@gmail.com (M.D.); helenakrysztofiak@gmail.com (H.K.); fsawczak@gmail.com (F.S.); szczechlamagdalena@gmail.com (M.S.); katarzyna.przytarska@gmail.com (K.P.); ewa.straburzynska-migaj@skpp.edu.pl (E.S.-M.); 2 Lord’s Transfiguration Clinical Hospital, Poznań University of Medical Sciences, 61-848 Poznan, Poland; 3 Faculty of Health Sciences, Wroclaw Medical University, 50-367 Wroclaw, Poland; marta.wleklik@umed.wroc.pl (M.W.); Izabella.uchmanowicz@umed.wroc.pl (I.U.)

**Keywords:** frailty syndrome, SHARE-FI, risk model, HFrEF

## Abstract

Frailty syndrome (FS) has recently attracted attention as one of the major predictors of heart failure (HF) course severity. We aimed to develop a simple tool for predicting frailty in hospitalized HF patients using routine clinical parameters. A total of 153 hospitalized patients diagnosed with heart failure with reduced ejection fraction (HFrEF) were included in the study. Presence of FS was assessed with the SHARE-FI questionnaire. Clinical and biochemical parameters were collected. Using ROC curves and logistic regression analysis, a model predicting FS presence was developed and tested. Proposed model includes five variables with following cut-off values (1 point for each variable): age > 50 years, systolic pressure on admission < 110 mmHg, total cholesterol < 4.85 mmol/L, bilirubin ≥ 15.5 mmol/L, and alanine aminotransferase ≤ 34 U/L. Receiving 5 points was considered a high risk of FS with positive and negative predictive values (NPV), 83% and 72%, respectively, and specificity of 97%. Awarding 2 points or less ruled out FS in the studied group with negative predictive value 94%. The presented novel, simple score predicts FS in HFrEF patients with routine clinical parameters and has good positive and negative predictive values.

## 1. Introduction

Heart failure (HF) is a growing global health problem affecting approximately 26 million people worldwide [1]. HF is associated with substantial morbidity and mortality, and it is an important cause of hospitalization in older adults [2]. Recently, frailty syndrome (FS) has attracted attention as a predictor of HF severity. Frailty is a biological and clinical syndrome characterized by a multidimensional and cumulative decline in many organs and systems [3]. It is an age-related syndrome characterized by decreased psychological reserve and resistance to stressors, with features of weakness, decreased endurance, and slowed performance [4]. According to Fried et al., the diagnosis of frailty is accurate when three of the following criteria are met: unintentional body weight loss, exhaustion, slow walking speed, weakness, and lowered physical activity [3]. Epidemiological data show that the estimated prevalence of frailty in the European population is about 18% [5]. Frail patients are at higher risk of adverse outcomes, including adverse drug reactions, prolonged hospitalizations, disability [6], and mortality, independently of and also in the absence of comorbidities [7]. In addition, frailty may accelerate the progression of chronic diseases, and chronic diseases may increase the risk of frailty or contribute to its faster development [8]. 

Several epidemiological studies have shown that HF is associated with frailty. Frailty rates in HF patients range from 15% to 79% [9,10]. Frailty impacts extubating time, in-patient length stay, and long-term mortality in advanced HF patients undergoing left ventricular assist device (LVAD) implantation [11]. Frail patients with HF are at risk of frequent rehospitalizations due to HF decompensation [12]. Over the last decade, the research regarding frailty in HF has significantly increased. In addition, the European Society of Cardiology (ESC) guidelines on heart failure recommended that healthcare professionals should monitor frailty in older patients [13]. International frailty guidelines recommend assessing frailty with validated instruments [14,15]. 

We aimed to develop a simple tool for predicting frailty in hospitalized HF patients using routine clinical parameters.

## 2. Materials and Methods

### 2.1. Study Population

This study included 153 patients with heart failure with reduced ejection fraction (HFrEF) who were hospitalized at the 1st Department of Cardiology of Poznan University of Medical Sciences. 

The inclusion criteria were: HFrEF history longer than three months; left ventricular ejection fraction (LVEF) < 40%, age ≥ 18 years, and signing the informed consent form. The study was conducted according to the guidelines of the Declaration of Helsinki, and the Ethics Committee of Poznan University of Medical Sciences approved it (No. 926/14). 

### 2.2. Analyzed Parameters

We diagnosed FS using the standardized Survey of Health, Ageing, and Retirement in Europe—Frailty Instrument (SHARE-FI), Munich Center for the Economics of Aging (MEA), Munich, Germany [16]. Clinical and echocardiographic findings were collected, including age, gender, comorbidities, prescribed medications, HF etiology, New York Heart Association (NYHA) functional class as indicated by ESC guidelines [13], body mass index (BMI), systolic blood pressure (SBP) on admission, diastolic blood pressure (DBP) on admission, heart rate (HR) at discharge, and left ventricular ejection fraction (LVEF). Moreover, the data on presence of atrial fibrillation as well as implantable cardiac devices were collected. 

Chronic kidney disease (CKD) was defined as an estimated glomerular filtration rate (eGFR) below 60 mL/min for three months or more. We calculated BMI using the formula BMI = weight (kg)/(height (m))^2^ and LVEF using the Simpson method [13,17]. 

Additionally, we evaluated the following laboratory parameters (collected at admission time) complete blood count, neutrophil to lymphocyte ratio (NLR), total iron-binding capacity (TIBC), serum sodium (Na^+^), potassium (K^+^), creatinine, B-type natriuretic peptide (BNP), N-terminal pro B-type natriuretic peptide (NT-proBNP), high-sensitivity C-reactive protein (hsCRP), total bilirubin (TBIL), alanine transaminase (ALT), gamma-glutamyltranspeptidase (GGTP), total cholesterol (CholT), triglycerides (TG), low-density lipoprotein (LDL), high-density lipoprotein (HDL), hemoglobin (Hb), serum iron, ferritin, transferrin saturation (TSAT), uric acid, fasting glucose, serum protein, and serum albumin. eGFR was calculated using both the Cockcroft–Gault (C-G) [18] and MDRD equation [19].

### 2.3. Diagnosing Frailty Syndrome

The frailty syndrome was diagnosed using the Polish version of the SHARE-FI questionnaire(https://sites.google.com/a/tcd.ie/share-frailty-instrument-calculators/translated-calculators last accessed on 17 December 2021). It was created and validated based on the first wave of the Survey of Health, Ageing, and Retirement in Europe to provide a simple tool facilitating the evaluation of frailty status [16]. This instrument is mainly recommended for screening frailty risk in the European population older than 50 years [16]. For Poland, the most reliable results were presented in patients over 60 years of age in out-patient as well as in-patient care [20]. SHARE-FI web calculator is available in different languages, and it is commonly used all over Europe. It consists of 5 variables: exhaustion (feeling of having too little energy to do things we want to do during the last month), loss of appetite, weakness, walking difficulties, and low physical activity [16]. We evaluated weakness by measuring twice patient’s maximal grip strength (in kilograms) with a manual dynamometer for both hands. Walking difficulties were defined as physical problems after walking 100 m or climbing one flight of stairs without resting, which lasted at least three months. The last question in the SHARE-FI questionnaire refers to a patient’s physical activity and concerns engaging in activities that require a low or moderate energy level (e.g., gardening, cleaning the car, or going on a walk). According to the SHARE-FI results, participants can be classified into three groups: (1) frail if their score is >3 for men and >2.13 for women; (2) pre-frail if the score is 1.21–3 for men and 0.32–2.13 for women; and (3) not frail if the score is <1.21 for men and <0.32 for women [16,21]. In our study, we combined the pre-frail and non-frail patients to simplify the analysis.

### 2.4. Statistical Analysis and Model Development

Continuous variables are presented as mean ± standard deviation (SD), while number (%) stands for categorical variables. According to the SHARE-FI questionnaire, we compared parameters between patients with diagnosed FS and those without FS. Mann—Whitney U test was used for continuous variables, and chi-square test with Yates correction, when needed, was used for categorical variables. 

For continuous variables that demonstrated differences in the Mann—Whitney U test, ROC (receiver operating characteristic) curve analysis was drawn for predicting FS presence. Optimal cut-off points with the highest Youden index value were chosen. Deriving cut-off points allowed to transform continuous parameters into categorical variables with values 0 or 1. For variables that were stimulants of the FS occurrence, 1 was assigned for values of the variable greater than or equal to the cut-off point and 0 for values less than the cut-off point. Similarly, for variables that were de-stimulants of the FS occurrence, 1 was assigned for values less than or equal to the cut-off point and 0 for values greater than the cut-off value obtained from ROC analysis.

Logistic regression was performed using FS presence as a dependent variable, while transformed and categorical variables, which revealed significant differences between the investigated groups, were used as categorical predictors. As a part of logistic regression, a univariable analysis was performed. We included all variables with *p* < 0.10 in the multivariable analysis. Using multivariable analysis with backward elimination, a frailty-predicting model was derived, and independent predictors of FS presence were specified. Depending on the obtained data, we aimed to simplify the model. ROC curve analysis was done for both the original and simplified model. ROC curves and their AUC (area under the curve) were compared between models to explore differences in predicting capabilities. On this basis, we determined the final shape of the model. Finally, we verified the effectiveness of the model. 

We considered *p* < 0.05 as statistically significant. Statistical analysis was done using Statistica 13.3 (Statsoft, now TIBCO, Palo Alto, CA, USA).

## 3. Results

### 3.1. Baseline Characteristics

Baseline characteristics are presented in Table 1. The study population mean age was 55.2 ± 11.6 years, and men stand for 81.7%. Ischemic heart disease as the HF cause was most frequent (48%). Most patients were classified in NYHA class III (46.4%). The mean LVEF was 24 ± 8.0%. FS was diagnosed in 34% of patients. Loop diuretics were used by 92.8% of patients, thiazides by 15%, ß-blockers by 97.4%, angiotensin-converting enzyme inhibitors (ACEI) or angiotensin receptor blockers (ARB) by 66%, angiotensin receptor-neprilysin inhibitors (ARNI) by 22.9%, mineralocorticoid receptor antagonists (MRA) by 85.6%, and statins by 60.8%. 

### 3.2. Frail and Non-Frail Subgroups

The variables demonstrated in Table 1 were compared between patients with FS (*n* = 52) and those without FS (*n* = 101) (combined non-frail and pre-frail groups) (Table 2).

### 3.3. ROC Curve Analysis

Age; systolic pressure on admission; presence of CKD; and presence of AF, BNP, NT-proBNP, eGFR C-G, total serum protein, albumin, bilirubin, ALT, total cholesterol, triglycerides, LDL, ferritin, TIBC, transferrin saturation, and serum iron were significantly different between the frail and non-frail patients, and they were chosen for model development. ROC curve analysis allowed the selection of optimal cut-off points for these variables to predict the frail status (Table 3). 

### 3.4. Regression Analysis and Model Development

We applied logistic regression to choose the independent predictors of the FS presence from these transformed variables and CKD and AF (Table 4). All variables mentioned in Table 3, except the serum protein ≤ 72.2 g/L, showed predicting capabilities for the FS presence with *p* < 0.10, and they were included in the multivariable analysis. The backward elimination method allowed independently eliminating variables not associated with the FS presence in the multivariable model (Table 5). Age ≥ 51 years, SBP ≤ 109 mmHg, TBIL ≥ 15.5µmol/L, CholT ≤ 4.85 mmol/L, and ALT ≤ 34 U/L were the independent predictors of FS (Table 6).

**Figure 1:**
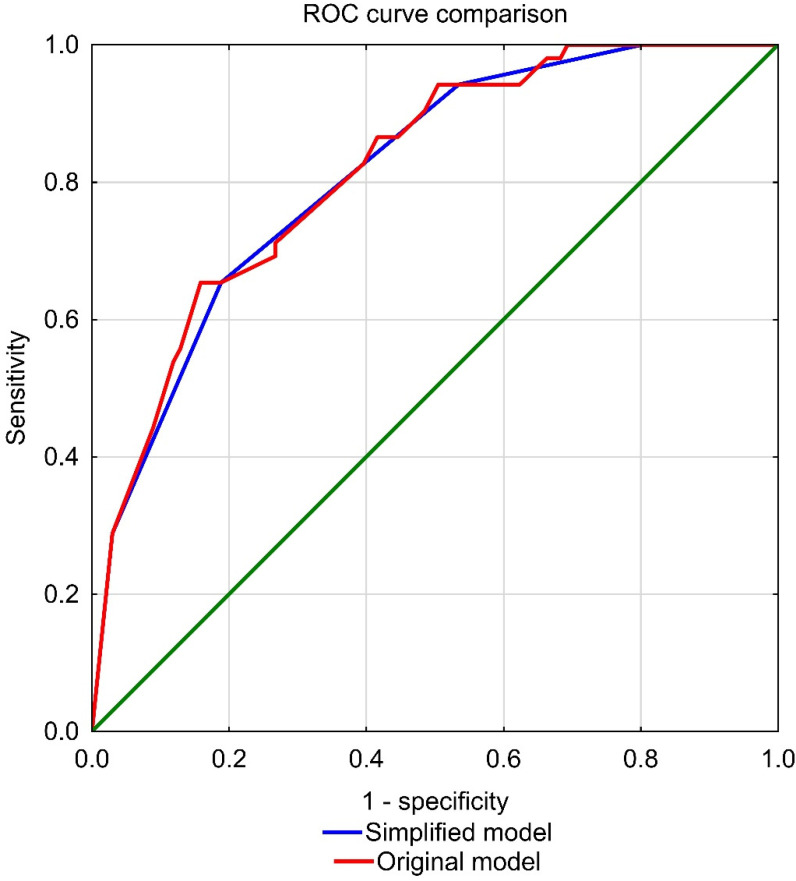
Visual comparison of ROC curves for the original and simplified model.

We decided to simplify the model by allocating 1 point for every parameter with a value equal to 1 (suggesting the presence of FS) instead of the β value in the original model. ROC curve analysis was used for each model to assess the predicting capabilities of the original and simplified models (Figure 1). We revealed no significant differences (*p* for ROC comparison = 0.65). AUC value for the original model was 0.818 (95% CI 0.750–0.885) and 0.813 (95% CI 0.745–0.881) for the simplified model. Hence, we chose the simplified model as the final model.

**Figure 2:**
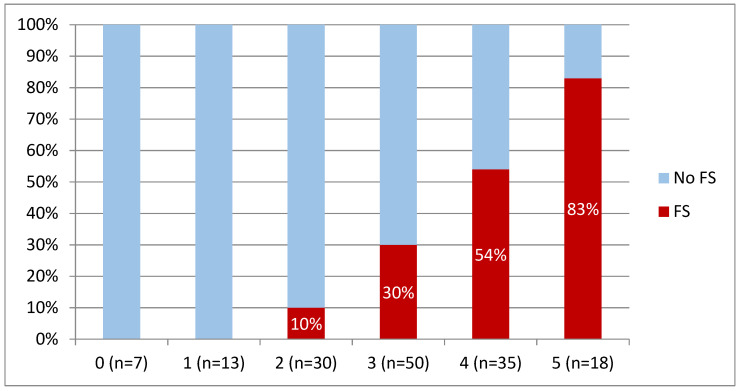
Presence of FS for a specified number of points in the simplified model. *n*—number of patients with specified number of points.

The simplified model was applied to divide patients into groups with a specified number of points (0, 1, 2, 3, 4, 5), and the presence of FS was assessed in each group (Figure 2). 

**Figure 3:**
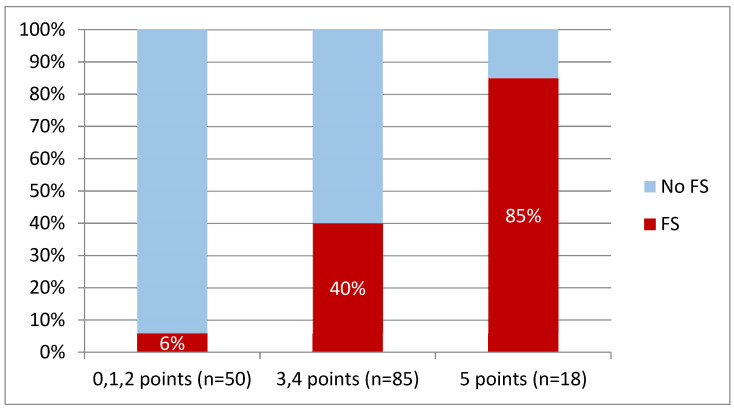
Presence of FS in groups with a different number of points, *n*—number of patients with specified number of points.

Sensitivity, specificity, PPV (positive predictive value), and NPV (negative predictive value) were counted for specified cut-off points of the model (≥1, ≥2, ≥3, ≥4, ≥5 points) (Table 7). Additionally, we divided patients into those with a low, intermediate, and high risk of frailty status (Figure 3). The final model is presented in Table 8.

Taking into account the fact that CholT was included in the final model, its predicting capabilities could be influenced by statin use. To assess that effect, statin use was added to the original model and in logistic regression it was insignificant; OR for statin use was 0.635 (95% CI 0.263–1.534), β = −0.455, and *p*-value 0.31. Meanwhile, relevance of CholT rose insignificantly (OR 3.921 (95% CI 1.135–13.546), *p* = 0.031 compared to OR 3.613 (1.047–12.465), and *p* = 0.042 without adjustment for statin therapy). Improvement in AUC was not statistically relevant: 0.824 (95% C.I. 0.757–0.891) for statin use involved vs. 0.818 (95% CI 0.750–0.885) for the original model; *p* for difference = 0.45. Due to lack of statistical confirmation that the addition of statin use could be beneficial, we decided not to involve it in the final model.

## 4. Discussion

We have proposed a novel model to predict FS in HF patients that includes five variables. We recommend the following cut-off values: age > 50 years, SBP on admission < 110 mmHg, total cholesterol < 4.85 mmol/L, bilirubin ≥ 15.5 mmol/L, and ALT ≤ 34 U/L. This model assumes awarding points for each variable exceeding the suggested cut-off value, which eventually efficiently discriminates the patients at risk of frailty. We propose considering patients at high risk of frailty if they exceed the cut-off values in every chosen variable. Such an approach shows good positive and negative predictive values, 83% and 72%, respectively. Awarding two points or less in this score seems to rule out FS in HF patients safely. Patients with 3–4 points, however, pose a borderline group worth further investigation.

The proposed model includes five variables that are readily available in every patient hospitalized due to HF. Thus, the model appears to be a simple and elegant tool very easy to apply in a busy clinical setting. Apart from its feasibility, it possesses two substantial advantages: low cost of assessing the proposed variables and their excellent availability on a patient’s examination or laboratory testing of blood samples at medical sites.

The development of the discussed model required diagnosing frailty in the included patients, which was done using the SHARE-FI questionnaire [16]. It is a simple validated tool and an alternative to Fried’s paradigm in the European context that offers a pre-calculated, population-representative, and gender-specific frailty class [16]. However, the SHARE-FI questionnaire is an elaborate version of Fried’s Frailty Phenotype [16], and as such, it shows a significant limitation, i.e., it evaluates only the clinical (physical) and functional domains of frailty (and even those only partially) [22]. The Heart Failure Association (HFA) position paper on frailty in HF patients suggests that the definition of frailty should not be narrowed down to the clinical domain, but the functional, social, and psychological domains should be included as well [22]. As of yet, there is no single tool to diagnose frailty comprehensively; hence, it seems justified to suspect that FS may be underdiagnosed among HF patients [22]. Still, even those diagnostic tools that use only the clinical and functional domains allow estimating the portion of HF patients with FS at 42.9% [23]. Furthermore, this way, frailty was shown to increase the risk of rehospitalizations and death in HF patients [16]. 

Apart from the relatively more straightforward tools mentioned above that concentrate on the clinical and functional domains of frailty, multidimensional tools aim to diagnose frailty using all four domains [24]. In general, they allow for higher estimates of the percentage of HF patients with FS, up to 47.4% [23]. It also seems that the more frailty domains are affected in patients, the worse are their prognoses [25]. However, these tools are considered too complex and time consuming to implement in everyday practice [22]. Our model allows identification of patients at risk of frailty using only age, SBP, and simple laboratory test results, therefore eliminating the need for broad, time-consuming screening of HF patients with more extensive questionnaires. The expected downside of our model, as being developed using the SHARE-FI questionnaire, would be a slightly lower sensitivity when compared with the multidimensional tools, but this seems unavoidable considering its simplicity.

In general, advanced age is not required to diagnose frailty among HF patients, and frailty has been observed among young adults [26,27] and children [28]. Nevertheless, our research shows that it is justified to use age as one of the discriminating variables and set its cut-off value for over 50 years because FS seems far less prevalent in younger patients. In addition, low SBP and elevated ALT can be associated both with the advanced HF and with frailty itself. However, the prevalence of frailty was demonstrated to correlate with NYHA class [29] regardless of LVEF [30,31].

SBP as a diagnostic parameter is recognized as a risk factor of cardiovascular events. According to the European Society of Cardiology (ESC) guidelines, there were no clinical trials evaluating BP targets and antihypertensive strategies in HFrEF [13]. BP targets should be personalized taking into account age and comorbidities. Primary drugs used in HFrEF decrease BP and should be used in full doses even when causing slight hypotension [13]. In our study, lower SBP (<110 mmHg) is recognized as a predictive factor of frailty. Patients with frailty have significantly lower SBP on admission compared with non-frailty patients (107.3 ± 19.8 mm Hg vs 115.7 ± 18.8 mm Hg, *p* = 0.007). Michael H. Lee et al. in their meta-analysis show that ambulatory HF patients with higher BP have better outcomes with a reduction in 7% mortality and 9% hospitalization by 10-mmHg increase in SBP [32], which confirms the validity of the proposed novel model to predict FS in HF patients in our study. Moreover, Concetta Di Nora et al. in their study debated the validity of lowering blood pressure in every case of a patient with HF [33], with the conclusion that in certain groups of patients, which include patients with HF and FS, one should avoid aggressive lowering of blood pressure is confirmed in our work.

The literature describes a phenomenon called the obesity paradox in heart failure patients, pointing out that obesity may decrease mortality in this specific group [34,35]. This observation is in direct contradiction to obesity known as a major cardiovascular risk factor and a cause of years of life lost. The cause of the paradox remains untracked; however, defining obesity solely by BMI value may lead to the omission of the role of abdominal obesity, which correlates with cardiovascular risk [36], and the mathematical conception of dividing weight and height gives no information about the distribution of fat in the body. In our study, there were no significant differences in BMI between frail patients compared to the non-frail (28.0 ± 5.5 vs. 29.1 ± 5.7 *p* = 0.15); therefore, BMI is not included in the proposed novel model to predict FS in HF patients. Due to the complexity of frailty syndrome, it may not be diagnosed according to low body weight but by a complex number of parameters influencing the diagnosis of FS and regarding the social and environmental functioning of the patient [3,37,38].

Ritt et al. demonstrated that frailty could be diagnosed with considerable accuracy using only laboratory parameters [39]. Their model was based on 22 routine blood parameters, including bilirubin and ALT. These two blood parameters proved to discriminate patients with frailty in our research, and to them, we added total cholesterol levels in our model. However, there is conflicting evidence regarding associations between cholesterol fraction concentrations and FS. Some authors showed that frailty might be associated with lower LDL blood concentrations [40,41]. On the other hand, some did not demonstrate significant differences in cholesterol fraction concentrations between patients with and without frailty [42,43,44]. We suppose the reason for this discrepancy might be some hidden bias. Most likely, the hypolipidemic medication used by patients included in various studies was not accounted for, or its influence could not be ruled out entirely by statistical methods.

In our analysis, the possibility of significant influence of statin therapy on the model based on CholT was excluded after adjustment for this parameter. Adding statin use insignificantly improved the model. Due to the methodology of the study, only parameters significant in multivariate analysis were subsumed into the model, and for that reason, statin use was rejected.

Low albumin concentration is a factor of bad prognosis in HF is connected with malnourishment and FS [45,46]. This parameter could be considered as an alternate one for CholT in our model; however, in the analysis, CholT had a better predicting value compared to albumin. Furthermore, CholT is low cost and easier to obtain for every patient.

In our model, we combined both the clinical and laboratory parameters, and to keep the model as simple as possible, we limited the number of variables to five. Similar attempts have been made, and it was demonstrated that even a model based on a relatively small number of variables could effectively predict FS [47]. This is important clinically because frail patients show an increased risk of rehospitalizations and death. This was demonstrated in numerous studies exploring the influence of frailty on the prognosis of HF patients in different clinical settings [12,48,49,50,51].

However, we must emphasize that the model we propose cannot be used to diagnose FS. Instead, this model aims to indicate patients at risk of frailty and thus requiring further diagnostics. The idea was to keep the model as simple as possible to make it easy to use in a busy clinical setting so that medical professionals would know which of their patients need special attention exceeding the usual pharmacological and non-pharmacological treatment (involving care in all four frailty domains).

### Limitations

The study’s main limitation is that the project is a single-center study and has a limited number of patients, which might limit the generalizability of the results. Second, due to the lack of a single FS assessment method dedicated to HF patients, we chose the SHARE-FI questionnaire, which was not validated in this patient group. Altogether, although our risk score should be further validated in other cohort groups, it summarizes the most recent and complete data on HFrEF patients suspected of having FS.

## 5. Conclusions

We propose a novel, simple score to predict FS in hospitalized HFrEF patients with good positive and negative predictive values. The score is based on routine clinical and laboratory parameters, and it can be easily applied in a busy clinical setting. Further longitudinal studies may show if the score can also be used to assess the prognosis of HFrEF patients.

## Figures and Tables

**Table 1:** Baseline characteristics.

Characteristics	Value ± SD
Age (years)	55.2 ± 11.6
Men	125 (81.7%)
BMI (kg/m^2^)	28.7 ± 5.6
HF of IHD etiology	73 (48.0%)
HF exacerbation	42 (27.8%)
SBP on admission (mmHg)	112.9 ± 19.5
DBP on admission (mmHg)	73.8 ± 12.6
HR on discharge (beats per minute)	73.9 ± 12.4
ICD	76 (49.7%)
CRT (CRT-P or CRT-D)	24 (15.7%)
Frailty status according to SHARE-FI	N (%)
Frail	52 (34.0)
Pre-frail	57 (37.2)
Non-frail	44 (28.8)
Comorbidities	N (%)
DM	42 (27.5)
COPD	14 (9.2)
CKD	26 (17.0)
Hypertension	79 (51.6)
Persistent or permanent AF	28 (18.3%)
NYHA class	N (%)
I	4 (2.6)
II	62 (40.5)
III	71 (46.4)
IV	16 (10.5)
I-II	66 (43.1)
III-IV	87 (56.9)
Biochemical parameters	Value ± SD
BNP (pg/mL)	690.6 ± 704.1
NT-proBNP (pg/mL)	3733 ± 5614
Uric acid (µmol/L)	469.9 ± 134.9
Creatinine (µmol/L)	105.4 ± 36.8
eGRF C-G (mL/min)	93.8 ± 41.9
eGFR MDRD (mL/min)	72.3 ± 24.3
Na^+^ (mmol/L)	139.1 ± 3.5
K^+^ (mmol/L)	4.31 ± 0.43
hsCRP (mg/L)	6.8 ± 10.1
Fasting glucose (mmol/L)	6.30 ± 1.74
Serum protein (g/L)	71.2 ± 7.4
Serum albumin (g/L)	40.5 ± 5.1
TBIL (µmol/L)	20.1 ± 12.2
ALT (U/L)	36.8 ± 22.3
GGT (U/L)	96.3 ± 105.3
CholT (mmol/L)	4.28 ± 1.16
TG (mmol/L)	1.57 ± 0.84
LDL (mmol/L)	2.62 ± 0.93
HDL (mmol/L)	1.18 ± 0.33
Hgb (mmol/L)	8.9 ± 1.1
Ferritin (μg/L)	162.9 ± 166.6
TIBC (μmol/L)	63.7 ± 11.7
TSAT (%)	25.5 ± 12.3
Serum iron (μmol/L)	15.9 ± 7.3
NLR	3.65 ± 1.89
Medications	N (%)
Loop diuretics	142 (92.8)
Thiazides	23 (15.0)
ß-blocker	149 (97.4)
ACEI/ARB	101 (66.0)
ARNI	35 (22.9)
MRA	131 (85.6)
Ca-blocker	7 (4.6)
Statin	93 (60.8)
Echocardiographic parameters	
LVEF (%)	24.0 ± 8.0
LVEDD (mm)	70.2 ± 10.6
RVD (mm)	37.2 ± 7.7
LAD (mm)	52.1 ± 10.6
IVS (mm)	10.2 ± 1.8
PW (mm)	10.1 ± 1.7
Ao (mm)	33.6 ± 4.7

BMI, body mass index; IHD, ischemic heart disease; SBP, systolic blood pressure; DBP, diastolic blood pressure; HR, heart rate; ICD, implantable cardioverter-defibrillator; CRT, cardiac resynchronization therapy; CRT-P, cardiac resynchronization therapy pacemaker; CRT-D, cardiac resynchronization therapy defibrillator; DM, diabetes mellitus; COPD, chronic obstructive pulmonary disease; CKD, chronic kidney disease; AF, atrial fibrillation; NYHA, New York Heart Association Classification; BNP, B-type natriuretic peptide; NT-proBNP, N-terminal pro B-type natriuretic peptide; eGFR, estimated glomerular filtration rate; C-G, Cockcroft–Gault equation; Na^+^, sodium concentration; K^+^, potassium concentration; hsCRP, high-sensitivity C-reactive protein; TBIL, total bilirubin; ALT, alanine transaminase; GGT, gamma-glutamyltranspeptidase; CholT, total cholesterol; TG, triglycerides; LDL, low-density lipoprotein; HDL, high-density lipoprotein; Hgb, hemoglobin; TIBC, total iron binding capacity; TSAT, transferrin saturation; NLR, neutrophil to lymphocyte ratio; ACEI, angiotensin converting enzyme inhibitor; ARB, angiotensin receptor blocker; ARNI, angiotensin receptor-neprilysin inhibitor; MRA, mineralocorticoid receptor antagonist; LVEF, left ventricular ejection fraction; LVEDD, left ventricular end-diastolic diameter; RVD, right ventricular diameter; LAD, left atrium diameter; IVS, interventricular septum thickness; PW, posterior wall of left ventricle; Ao, aorta.

**Table 2:** Differences in chosen parameters in frail and non-frail patients based on the SHARE-FI questionnaire.

Characteristics	Frail Syndrome (*n* = 52)	No Frail Syndrome (*n* = 101)	*p*
Age (years)	59.1 ± 8.6	53.1 ± 12.4	0.002 *
Men	41 (78.9%)	84 (83.2%)	0.51
BMI (kg/m^2^)	28.0 ± 5.5	29.1 ± 5.7	0.15
IHD etiology	28 (53.8%)	46 (45.5%)	0.33
HF exacerbation	21 (40.4%)	21 (20.8%)	0.01
SBP on admission (mmHg)	107.3 ± 19.8	115.7 ± 18.8	0.007 *
DBP on admission (mmHg)	72.3 ± 12.0	74.6 ± 12.9	0.07
HR on discharge (beats per minute)	76.1 ± 13.6	72.7 ± 11.7	0.17
ICD	25 (48.1%)	51 (50.5%)	0.78
CRT (CRT-P or CRT-D)	10 (19.2%)	14 (13.9%)	0.38
Comorbidities			
DM	12 (23.1%)	30 (29.7%)	0.38
COPD	4 (7.7%)	10 (9.9%)	0.88
CKD	14 (26.9%)	12 (11.9%)	0.02 *
Hypertension	27 (51.9%)	52 (51.5%)	0.96
Persistent or permanent AF	15 (28.8%)	13 (12.9%)	0.015 *
Biochemical parameters			
BNP (pg/mL)	851.9 ± 698.7	604.2 ± 696.5	0.009 *
NT-proBNP (pg/mL)	5216.0 ± 6314.9	3055.5 ± 5170.4	0.0006 *
Uric acid (µmol/L)	472.8 ± 124.0	468.4 ± 141.0	0.77
Creatinine (µmol/L)	109.6 ± 39.3	103.2 ± 35.4	0.18
eGFR C-G (mL/min)	81.5 ± 35.4	100.2 ± 43.7	0.004 *
eGFR MDRD (mL/min)	67.3 ± 22.7	74.9 ± 24.9	0.053
Na^+^ (mmol/L)	138.5 ± 3.5	139.4 ± 3.5	0.13
K^+^ (mmol/L)	4.24 ± 0.46	4.34 ± 0.40	0.21
hsCRP (mg/L)	7.5 ± 9.5	6.4 ± 10.4	0.24
Fasting glucose (mmol/L)	6.09 ± 1.05	6.42 ± 2.01	0.97
Serum protein (g/L)	69.0 ± 8.5	72.4 ± 6.5	0.02 *
Serum albumin (g/L)	39.6 ± 5.8	41.0 ± 4.6	0.03 *
TBIL (µmol/L)	24.0 ± 13.3	18.0 ± 11.0	0.0009 *
ALT (U/L)	30.7 ± 17.4	39.9 ± 23.9	0.008 *
GGT (U/L)	108.7 ± 92.1	89.8 ± 111.6	0.08
CholT (mmol/L)	3.76 ± 0.88	4.55 ± 1.20	0.0004 *
TG (mmol/L)	1.32 ± 0.65	1.70 ± 0.90	0.006 *
LDL (mmol/L)	2.29 ± 0.79	2.79 ± 0.96	0.004 *
HDL (mmol/L)	1.11 ± 0.31	1.21 ± 0.33	0.15
Hgb (mmol/L)	8.75 ± 1.20	9.02 ± 1.07	0.17
Ferritin (μg/L)	127.1 ± 130.6	101.6 ± 180.5	0.004 *
TIBC (μmol/L)	66.3 ± 13.2	62.3 ± 10.7	0.02 *
TSAT (%)	21.3 ± 12.3	27.6 ± 11.8	0.002 *
Serum iron (μmol/L)	14.0 ± 7.7	16.9 ± 7.0	0.009 *
NLR	3.97 ± 2.14	3.48 ± 1.74	0.13
Medications			
Loop diuretics	52 (100.0%)	90 (89.1%)	0.03
Thiazides	11 (21.2%)	12 (11.9%)	0.13
ß-blocker	51 (98.1%)	98 (97.0%)	0.88
ACEI/ARB	37 (71.2%)	64 (63.4%)	0.34
ARNI	9 (17.3%)	26 (25.7%)	0.33
MRA	48 (92.3%)	83 (82.2%)	0.15
Ca-blocker	2 (3.9%)	5 (5.0%)	0.92
Statin	34 (65.4%)	59 (58.4%)	0.40
Echocardiographic parameters			
LVEF (%)	22.3 ± 8.1	24.9 ± 7.8	0.05
LVEDD (mm)	69.8 ± 11.6	70.4 ± 10.0	0.62
RVD (mm)	36.7 ± 6.9	37.4 ± 8.1	0.99
LAD (mm)	55.2 ± 10.2	50.5 ± 10.5	0.02 *
IVS (mm)	10.4 ± 2.1	10.1 ± 1.6	0.60
PW (mm)	10.2 ± 2.2	10.0 ± 1.4	0.95
Aorta (mm)	33.1 ± 4.4	33.9 ± 4.9	0.32

* variables included into the further analysis as potential markers predicting frailty, BMI, body mass index; IHD, ischemic heart disease; SBP, systolic blood pressure; DBP, diastolic blood pressure; HR, heart rate; ICD, implantable cardioverter-defibrillator; CRT, cardiac resynchronization therapy; CRT-P, cardiac resynchronization therapy pacemaker; CRT-D, cardiac resynchronization therapy defibrillator; DM, diabetes mellitus; COPD, chronic obstructive pulmonary disease; CKD, chronic kidney disease; AF, atrial fibrillation; BNP, B-type natriuretic peptide; NT-proBNP, N-terminal pro B-type natriuretic peptide; eGFR, estimated glomerular filtration rate; C-G, Cockcroft–Gault equation; Na^+^, sodium concentration; K ^+^, potassium concentration; hsCRP, high-sensitivity C-reactive protein; TBIL, total bilirubin; ALT, alanine transaminase; GGTP, gamma-glutamyltranspeptidase; CholT, total cholesterol; TG, triglycerides; LDL, low-density lipoprotein; HDL, high-density lipoprotein; Hgb, hemoglobin; TIBC, total iron binding capacity; TSAT, transferrin saturation; NLR, neutrophil to lymphocyte ratio; ACEI, angiotensin converting enzyme inhibitor; ARB, angiotensin receptor blocker; ARNI, angiotensin receptor-neprilysin inhibitor; MRA, mineralocorticoid receptor antagonist; LVEF, left ventricular ejection fraction; LVEDD, left ventricular end-diastolic diameter; RVD, right ventricular diameter; LAD, left atrium diameter; IVS, interventricular septum thickness; PW, posterior wall of left ventricle; Ao, aorta. * variables included in the univariable analysis of logistic regression.

**Table 3:** Parameters with significant differences between the frail and non-frail patients and their optimal cut-off points for predicting frailty syndrome derived from analysis of ROC curves.

Characteristics	Cut-Off Points	Association with the FS Presence	Transformed Variable for Frailty Prediction
Continuous variables			
Age (years)	51	Stimulus	age ≥ 51
SBP on admission (mmHg)	109	De-stimulus	SBP ≤ 109 mmHg
BNP (pg/mL)	209.6	Stimulus	BNP ≥ 209.6 pg/mL
NT-proBNP (pg/mL)	1623	Stimulus	NT-proBNP ≥ 1623 pg/mL
TSAT (%)	22.4	De-stimulus	TSAT ≤ 22.4%
Serum iron (μmol/L)	13.0	De-stimulus	serum iron ≤ 13 qmol/L
Ferritin (ng/mL)	132.3	De-stimulus	ferritin ≤ 132.3 ng/mL
Serum protein (g/L)	72.2	De-stimulus	serum protein ≤ 72.2 g/L
Serum albumin (g/L)	37.9	De-stimulus	serum albumin ≤ 37.9 g/L
TBIL (µmol/L)	15.5	Stimulus	TBIL ≥ 15.5µmol/L
ALT (U/L)	34	De-stimulus	ALT ≤ 34 U/L
CholT (mmol/L)	4.85	De-stimulus	CholT ≤ 4.85 mmol/L
TG (mmol/L)	1.25	De-stimulus	TG ≤ 1.25 mmol/L
LDL (mmol/L)	2.68	De-stimulus	LDL ≤ 2.68 mmol/L
LAD (mm)	58	Stimulus	LAD ≥ 58 mm
Categorical variables			
CKD	-	Stimulus	-
AF (persistant or permanent)	-	Stimulus	-

SBP, systolic blood pressure; BNP, B-type natriuretic peptide; NT-proBNP, N-terminal pro B-type natriuretic peptide; TSAT, transferrin saturation; TBIL, total bilirubin; ALT, alanine transaminase; CholT, total cholesterol; TG, triglycerides; LDL, low-density lipoprotein; LAD, left atrium diameter; CKD, chronic kidney disease; AF, atrial fibrillation.

**Table 4:** The univariable analysis with the FS presence as the dependent variable and transformed variables as categorical predictors.

Characteristics	Estimated β (95% CI)	Wald.	OR (95% CI)	*p*
Age ≥ 51	1.74 (0.73–2.74)	11.4	5.67 (2.07–15.51)	0.0007 *
SBP ≤ 109 mmHg	1.08 (0.38–1.77)	9.34	2.94 (1.47–5.87)	0.002 *
BNP ≥ 209.6 pg/mL	0.95 (0.24–1.66)	6.94	2.59 (1.27–5.24)	0.008 *
NT-proBNP ≥ 1623 pg/mL	0.94 (0.25–1.63)	7.08	2.56 (1.28–5.1)	0.008 *
eGFR C-G ≤ 91.1 mL/min	1.38 (0.63–2.14)	12.9	3.99 (1.87–8.48)	0.0003 *
TSAT ≤ 22.4%	1.10 (0.40–1.80)	9.64	3.00 (1.50–6.02)	0.002 *
Serum iron ≤ 13 μmol/L	0.86 (0.18–1.54)	6.06	2.36 (1.19–4.67)	0.01 *
Ferritin ≤ 132.3 ng/mL	1.10 (0.37–1.82)	8.76	3.00 (1.45–6.20)	0.003 *
Serum protein ≤ 72.2 g/L	0.44 (−0.28–1.16)	1.42	1.55 (0.75–3.19)	0.23
Serum albumin ≤ 37.9 g/L	0.65 (−0.02–1.33)	3.58	1.92 (0.98–3.78)	0.06 *
TBIL ≥ 15.5 µmol/L	1.32 (0.60–2.04)	13.0	3.76 (1.83–7.73)	0.0003 *
ALT ≤ 34 U/L	1.22 (0.47–1.98)	10.1	3.40 (1.60–7.23)	0.0015 *
CholT ≤ 4.85 mmol/L	1.85 (0.75–2.95)	10.9	6.36 (2.12–19.1)	0.001 *
TG ≤ 1.25 mmol/L	0.73 (0.05–1.41)	4.41	2.68 (1.26–5.70)	0.04 *
LDL ≤ 2.68 mmol/L	0.98 (0.23–1.74)	6.54	2.07 (1.05–4.10)	0.01 *
LAD ≥ 58 mm	1.15 (0.41–1.90)	9.24	3.16 (1.51–6.65)	0.02 *
CKD	1.00 (0.15–1.86)	5.25	2.732 (1.16–6.45)	0.02 *
AF (persistent or permanent)	1.01 (0.17–1.85)	5.60	2.744 (1.19–6.33)	0.02 *

* variables included into the further analysis as potential markers predicting frailty; SBP, systolic blood pressure; BNP, B-type natriuretic peptide; NT-proBNP, N-terminal pro B-type natriuretic peptide; GFR, glomerular filtration rate; C-G, Cockcroft–Gault equation; TSAT, transferrin saturation; TBIL, total bilirubin; ALT, alanine transaminase; CholT, total cholesterol; TG, triglycerides; LDL, low-density lipoprotein; LAD, left atrium diameter; CKD, chronic kidney disease; AF, atrial fibrillation.

**Table 5:** The frailty-predicting model using the backward elimination method.

Step	Parameters Included in the Model	Removed Parameter	*p* of Elimination
1.	ALL	TSAT ≤ 22.4%	0.81
2.	ALL except TSAT ≤ 22.4%	AF	0.78
3.	ALL except TSAT ≤ 22.4%, AF	CKD	0.69
4.	ALL except TSAT ≤ 22.4%, AF, CKD	NT-proBNP ≥ 1623 pg/mL	0.70
5.	ALL except TSAT ≤ 22.4%, AF, CKD, NT-proBNP ≥ 1623 pg/mL	serum iron ≤ 13 qmol/L	0.50
6.	ALL except TSAT ≤ 22.4%, AF, CKD, NT-proBNP ≥ 1623 pg/mL	LDL ≤ 2.68 mmol/L	0.43
7.	ALL except TSAT ≤ 22.4%, AF, CKD, NT-proBNP ≥ 1623 pg/mL, LDL ≤ 2.68 mmol/L	TG ≤ 1.25 mmol/L	0.51
8.	ALL except TSAT ≤ 22.4%, AF, CKD, NT-proBNP ≥ 1623 pg/mL, LDL ≤ 2.68 mmol/L, TG ≤ 1.25 mmol/L	LAD ≥ 58 mm	0.33
9.	ALL except TSAT ≤ 22.4%, AF, CKD, NT-proBNP ≥ 1623 pg/mL, LDL ≤ 2.68 mmol/L, TG ≤ 1.25 mmol/L, LAD ≥ 58 mm	ferritin ≤ 132.3 ng/mL	0.25
10.	age ≥ 51, SBP ≤ 109 mmHg, BNP ≥ 209.6 pg/mL, serum albumin ≤ 37.9 g/L, TBIL ≥ 15.5µmol/L, ALT ≤ 34 U/L, CholT ≤ 4.85 mmol/L	serum albumin ≤ 37.9 g/L	0.13
11.	age ≥ 51, SBP ≤ 109 mmHg, BNP ≥ 209.6 pg/mL, TBIL ≥ 15.5 µmol/L, ALT ≤ 34 U/L, CholT ≤ 4.85 mmol/L,	BNP ≥ 209.6 pg/mL	0.06
12.	age ≥ 51, SBP ≤ 109 mmHg, TBIL ≥ 15.5 µmol/L, ALT ≤ 34 U/L, CholT ≤ 4.85 mmol/L	-	-

ALL = age ≥ 51, SBP ≤ 109 mmHg, BNP ≥ 209.6 pg/mL, NT-proBNP ≥ 1623 pg/mL, eGFR C-G ≤ 91.1 mL/min, TSAT ≤ 22.4%, serum iron ≤ 13 qmol/L, ferritin ≤ 132.3 ng/mL, serum albumin ≤ 37.9 g/L, TBIL ≥ 15.5 µmol/L, ALT ≤ 34 U/L, CholT ≤ 4.85 mmol/L, TG ≤ 1.25 mmol/L, LDL ≤ 2.68 mmol/L, LAD ≥ 58 mm, CKD, AF.

**Table 6:** The frailty-predicting model based on the results of logistic regression.

Characteristics	Estimated β (95% CI)	Wald.	OR (95% CI)	*p*
Intercept	−5.045 (−6.283–(−3.267))	30.927	0.006 (0.001–0.038)	<0.0001
age ≥ 51	1.757 (0.641–2.873)	9.517	5.794 (1.898–17.689)	0.002
SBP ≤ 109 mmHg	1.026 (0.210–1.841)	6.078	2.789 (1.234–6.303)	0.014
TBIL ≥ 15.5 µmol/L	1.312 (0.470–2.155)	9.135	3.715 (1.599–8.628)	0.002
ALT ≤ 34 U/L	1.177 (0.300–2.054)	6.923	3.245 (1.350–7.799)	0.009
CholT ≤ 4.85 mmol/L	1.285 (0.046–2.523)	4.134	3.613 (1.047–12.465)	0.042

SBP, systolic blood pressure; TBIL, total bilirubin; ALT, alanine transaminase; CholT, total cholesterol.

**Table 7:** Sensitivity, specificity, positive predictive value, and negative predictive value for specified cut-off points.

Cut-Off Value (Value Higher or Equal Indicates FS Presence)	Sensitivity	Specificity	PPV	NPV
5 points	0.288	0.970	0.833	0.726
4 points	0.654	0.812	0.642	0.820
3 points	0.942	0.465	0.476	0.940
2 points	1.000	0.198	0.391	1.000
1 point	1.000	0.069	0.356	1.000

PPV, positive predictive value; NPV, negative predictive value.

**Table 8:** The components of the final model.

Paramete	The Cut-Off Value for Predicting Frailty	Points
Age	higher or equal to 51 years	1
SBP on admission	lower or equal to 109 mmHg	1
CholT	lower or equal to 4.85 mmol/L	1
TBIL	higher or equal to 15.5 μmol/L	1
ALT	lower or equal to 34 U/L	1

SBP, systolic blood pressure; CholT, total cholesterol; TBIL, total bilirubin; ALT, alanine transaminase.

## Data Availability

The data presented in this study are available on request from the corresponding author.
